# Supplementary material for: Evidence supported by Mendelian randomization: impact on inflammatory factors in knee osteoarthritis
Source: Front Med (Lausanne). 2024 May 28;11:1382836. doi: 10.3389/fmed.2024.1382836 (PMC11165061; doi:10.3389/fmed.2024.1382836)
Supplement: Supplementary file 1 [file Data_Sheet_1.ZIP › data/GCST90274819_5e6/819.leaveoneout.pdf]

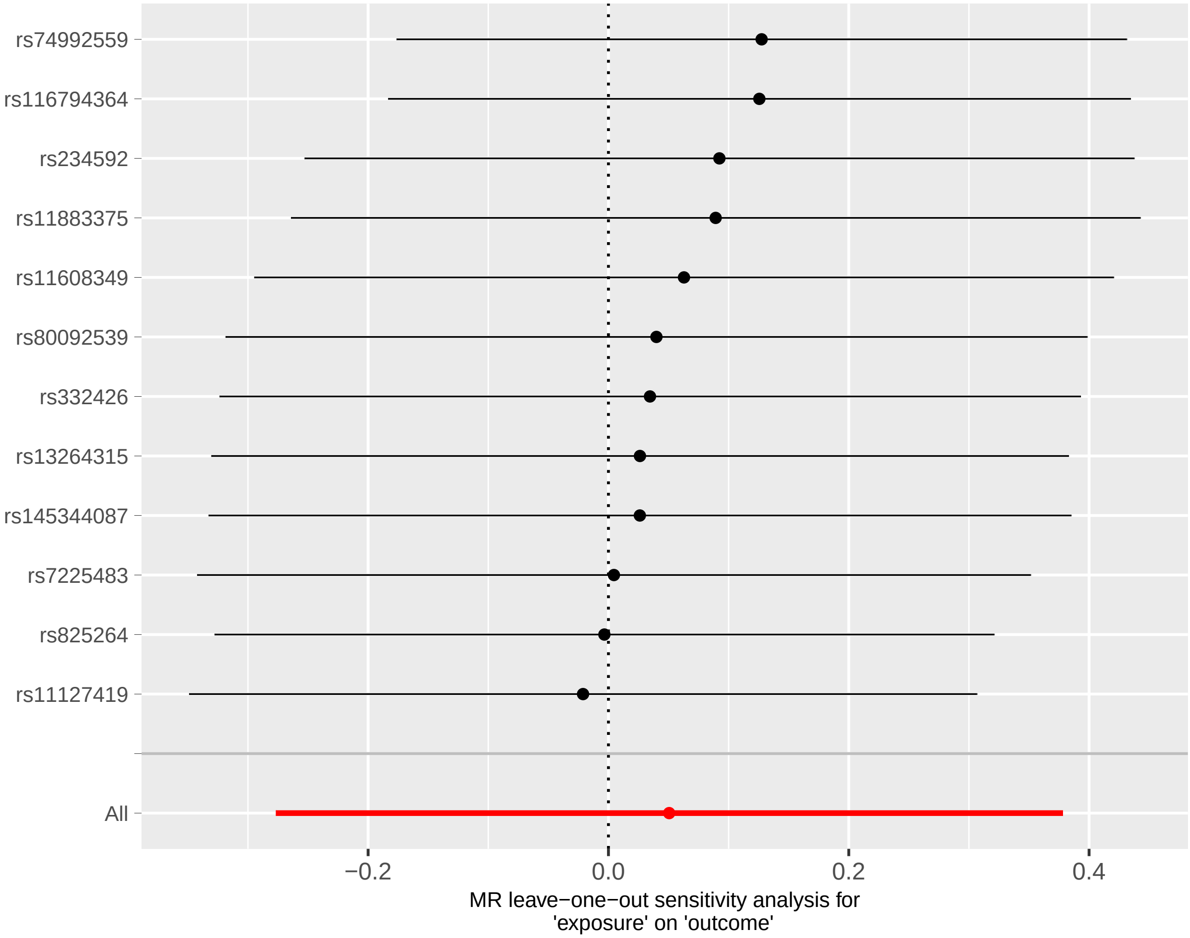
rs74992559
rs116794364
rs234592
rs11883375
rs11608349
rs80092539
rs332426
rs13264315
rs145344087
rs7225483
rs825264
rs11127419
All
−0.2
0.0
0.2
0.4
MR leave−one−out sensitivity analysis for
'exposure' on 'outcome'
